# Structure–Activity Relationship of Graphene-Based Materials: Impact of the Surface Chemistry, Surface Specific Area and Lateral Size on Their In Vitro Toxicity

**DOI:** 10.3390/nano11112963

**Published:** 2021-11-04

**Authors:** Salma Achawi, Bruno Feneon, Jérémie Pourchez, Valérie Forest

**Affiliations:** 1 Mines Saint-Etienne, Univ. Lyon, Univ. Jean Monnet, Etablissement Français du Sang, INSERM, U1059 Sainbiose, Centre CIS, 42023 Saint-Etienne, France; salma.achawi@emse.fr (S.A.); pourchez@emse.fr (J.P.); 2 Manufacture Française des Pneumatiques Michelin, Place des Carmes Déchaux, CEDEX 9, 63040 Clermont-Ferrand, France; bruno.feneon@michelin.com; 3 Valérie Forest, Mines Saint-Etienne, 158 Cours Fauriel, CS 62362, CEDEX 2, 42023 Saint-Etienne, France

**Keywords:** graphene-based materials, structure–activity relationship, toxicity, safe-by-design

## Abstract

Predictive toxicity and structure–activity relationships (SARs) are raising interest since the number of nanomaterials has become unmanageable to assess their toxicity with a classical case-by-case approach. Graphene-based materials (GBMs) are among the most promising nanomaterials of this decade and their application might lead to several innovations. However, their toxicity impact needs to be thoroughly assessed. In this regard, we conducted a study on 22 GBMs to investigate their potential SARs by performing a complete physicochemical characterization and in vitro toxicity assessment (on RAW264.7 cells). We used GBMs of variable lateral size (0.5–38 µm), specific surface area (SSA, 30–880 m²/g), and surface oxidation (2–17%). We observed that reduced graphene oxides (RGOs) were more reactive than graphene nanoplatelets (GNPs), potentially highlighting the role of GBM’s surface chemistry and surface defects density in their biological impact. We also observed that for GNPs, a smaller lateral size caused higher cytotoxicity. Lastly, GBMs showing a SSA higher than 200 m²/g were found to induce a higher ROS production. Mechanistic explanations are proposed in the discussion. In conclusion, pairing a full physicochemical characterization with a standardized toxicity assessment of a large set of samples allowed us to clarify SARs and provide an additional step toward safe-by-design GBMs.

## 1. Introduction

The nanotoxicology field emerged almost 20 years ago [1] and the number of nanomaterials has exponentially increased ever since [2,3]. Many nanomaterials have interesting potential in various industrial fields such as electronics [4], optics [5] but also biomedical [6]. Most of these applications are not achieved yet due to the potential hazard of these materials which cause many concerns, especially for occupational exposure [7,8]. Hence, assessing nanomaterials’ risk is not only an absolute necessity for public health but could also lead to numerous avenues of potential scientific and industrial progress. The risk assessment is composed of two major steps: exposure and hazard characterization [9]. In this work, we will focus on hazard assessment.

The hazard assessment for nanomaterials can vary depending on the country. It is yet safe to state that in vivo testing is often required, especially in the case of occupational exposure. A dramatically huge number of nanomaterials are available and assessing their toxicity on a case-by-case basis is impossible, as it would be too expensive and time-consuming. Moreover, many scientists try to reduce the use of animal testing and focus on alternative approaches when in vivo testing is not essential [10]. These alternative approaches emerged in the past decade, including grouping [11] or read across [12]. The study of structure–activity relationships (SARs) is another method that opens new perspectives and is now considered as a relevant alternative method for regulatory purposes [13]. Nanomaterials’ toxicity depends on their physicochemical characteristics [14]. In particular, size, distribution, agglomeration state, shape, crystal structure, chemical composition, surface area, surface chemistry, surface charge, and porosity are of paramount importance [15]. Knowing which physicochemical characteristics can impact a specific biological endpoint and how would be a first step toward safe-by-design nanomaterials [16,17].

Graphene-based materials (GBMs) were isolated in 2004 [18] and are raising a lot of attention since. As for other nanomaterials, their application potential is very important and they could lead to numerous innovations including batteries [19,20], electronics [21], and even electronic skin [22]. GBMs include various materials, all derived from graphene such as graphene oxide (GO), reduced graphene oxide (RGO), graphene nanoplatelet (GNP), or graphene quantum dot (GQD). These materials can vary by their size, oxidation state, or thickness [23].

GBMs’ potential toxicity is not perfectly clear yet. While some GBMs appear quite poorly toxic, others show very strong signs of toxicity. Inflammation [24] and cytotoxicity [25] were also highlighted for some GBMs. GBM toxicity is known to vary depending on their size [26], shape [27], or oxidation state [28] for example. However, these SARs are particularly complex, and the available literature does not currently allow us to have a clear overview of them due to a lack of both physicochemical characterization and standardized toxicity assays [29].

In this paper, we propose to explore the potential structure–activity relationships of GBMs using 22 samples. We fully characterized the physicochemical features of all these nanomaterials and explored their in vitro toxicity (cytotoxicity, pro-inflammatory response, and oxidative stress). We then investigated potential correlations between the physicochemical features and toxicity endpoints that could highlight a potential structure–activity relationship.

## 2. Materials and Methods

### 2.1. Nanomaterials and Physicochemical Characterization

These tested samples are all industrial, commercial materials that were obtained from five different suppliers. They include a total of 22 GBMs: 15 GNPs and 7 RGOs. Their specific surface area was determined with the BET technique (adsorption of nitrogen, with degassing system Micromeritics, Norcross, GA, USA). Their surface oxidation was determined with XPS (X-ray Photo Spectroscopy, Quantera Scanning XPS microprobe, Physical Electronics, Chanhassen, MN, USA). Lateral size was determined with electronic microscopy (Field Emission Scanning Electron Microscope, from JEOL, Tokyo, Japan). Surface defects were estimated with the ID/IG intensity ratio, calculated with Raman spectroscopy (XploRA, Horiba Scientific, Kyoto, Japan). For comparison, we also tested two samples of carbon black and one sample of amorphous silica.

### 2.2. Toxicity Assessment

#### 2.2.1. Cell Culture

The RAW 264.7 murine macrophage cell line was provided by ATCC Cell Biology Collection (Promochem LGC, Teddington, UK). It was derived from mice peritoneal macrophages transformed by the Albeson Murine Leukemia Virus. The rationale behind the choice of this cellular model is based on the fact that macrophages are ubiquitous cells, part of the immune system and thus they act as the first responders upon exposure to nanomaterials [30]. They are responsible for the recognition and elimination of foreign bodies through phagocytosis. Cells were grown in Dulbecco’s Modified Eagle Medium (DMEM, Invitrogen, Waltham, MA, USA) supplemented with 10% fetal calf serum and 1% penicillin-streptomycin (Sigma-Aldrich, Darmstadt, Germany) (complete DMEM, cDMEM) and maintained at 37 °C under a 5% carbon dioxide humidified atmosphere. Cells were seeded in 96 wells plates the day before exposure.

#### 2.2.2. Cell Exposure to Nanomaterials

This exposure protocol was used for LDH, TNF-α, and DCFH-DA assays. A high concentrated (1600 µg/mL) stock solution of nanomaterials was prepared in deionized water. This stock solution was dispersed with a vortex, and a two-step sonication as described below:

- Vortex TopMix FB15024 Fisher Scientific (Hampton, VA, USA)—Mode: continuous—Frequency: 40 Hz—Room temperature—Time: 60 s.
- Ultrasonicate Bath Fisher Scientific Bioblock (Hampton, VA, USA)—Temperature: 20 °C—Frequency: 130 Hz—Power: 100%—Time: 15 min.
- Branson S-450 Sonicator, without probe (Emerson, Saint Louis, MO, USA)—Program: 2 s pulse + 2 s inter—70% for 10 min then 85% for 5 min.

We then diluted the stock solutions in cDMEM to reach final exposure concentrations of 15, 30, 60, and 120 µg/mL, as recommended for the in vitro assessment of nanomaterial hazard [31]. We added these solutions to the RAW264.7 cells.

#### 2.2.3. Cytotoxicity

To evaluate cell membrane integrity, the cellular release in the supernatant of cytoplasmic lactate dehydrogenase (LDH) was assessed using the CytoTox-96™ Homogeneous Membrane Integrity Assay (Promega, Charbonnières-les-Bains, France) according to the manufacturer’s instructions. The optical density of the samples was determined using a microplate reader (Multiskan RC; Thermolabsystems, Helsinki, Finland) set to 450 nm. Three independent experiments were performed, each in quadruplicate and the activity of the released LDH was reported to that of negative control cells (incubated without nanoparticles). A positive control consisted of the maximal cellular LDH released after cells lysis.

#### 2.2.4. Pro-Inflammatory Response

After incubation with nanoparticles, the production of TNF-α was assessed in the supernatant using a commercial ELISA Kit (Quantikine^®^ Mouse TNF-α Immunoassay; R&D Systems, Lille, France) according to the manufacturer’s instructions. The optical density of each sample was determined using a microplate reader (Multiskan RC; Thermolabsystems, Helsinki, Finland) set to 450 nm. A standard curve was established, and results were expressed in picograms of TNF-α per milliliter of supernatant. Three independent experiments were performed, each in quadruplicate, and the production of TNF-α was reported to that of control cells (incubated without nanoparticles).

#### 2.2.5. ROS Production

A large array of ROS activity can be assessed with the OxiSelect™ ROS Assay Kit (Euromedex, Mundolsheim, France). The assay uses the conversion of a non-fluorescent substrate, 2.7′-dichlorodihydrofluorescein diacetate that can easily diffuse through cell membranes and be converted into a fluorogenic molecule 2′.7′-dichlorodihydrofluorescein (DCF) in presence of ROS: fluorescence amount is directly related to ROS level. Fluorescence was detected using a Fluoroskan Ascent fluorometer (Ex: 480 nm, Em: 530 nm, Thermolabsystems, Helsinki, Finland) after a 90 min or 24 h incubation of cells with the nanoparticles. A positive control was included incubating cells with H_2_O_2_ (1 mM). Three independent experiments were performed, each in duplicate, and the generation of ROS was reported to that of the negative control (cells incubated without nanoparticles).

#### 2.2.6. FRAS Measurement

The FRAS (ferric reducing ability of the serum) assay measures the biological oxidative damage of the nanomaterials on blood human serum. Briefly, it is an acellular assay that measures the capacity of a serum sample that has been exposed to nanomaterials (or any other chemicals) to reduce ferric ions to ferrous ions. The reducing capacity of a biological matrix can then approximate its antioxidant capacity [32]. This assay is described elsewhere [33] and work was recently done by our team to study the feasibility of this test on GBMs, where a complete optimized protocol is described [34].

#### 2.2.7. Toxicity Classification

For the LDH, TNF-α, and ROS assays, a toxicity classification was performed as follows:

- No toxicity: no significant response compared to negative control even at the highest dose (120 µg/mL).
- High LOAEL (Lowest Observed Adverse Effect Level): significant response compared to negative control when the exposure dose is high: 120 µg/mL or 60 µg/mL. Please note that for simplification purposes, this class is referred to as “moderate toxicity”.
- Low LOAEL: significant response compared to negative control when the exposure dose is low: 15 µg/mL or 30 µg/mL. Please note that for simplification purposes, this class is referred to as “high toxicity”.

We then compared samples that caused no toxicity to samples that caused toxicity (grouping moderate and high toxicity).

Please note that for the FRAS assay, the toxicity classification was realized differently since this assay represents a different approach and uses different exposure concentrations.

- Low FRAS effect was declared when the response at the lowest exposure dose (5 g/L) did not exceed 30 mMTEU.
- Moderate FRAS effect was declared when the response at the lowest exposure dose (5 g/L) was between 30 and 60 mMTEU.
- High FRAS effect was declared when the response at the lower exposure (5 g/L) exceeded 60 mMTEU.

## 3. Results

### 3.1. Physicochemical Characterization of the Nanomaterials

The main physicochemical features of the tested samples are reported in Table 1. Results from the Inductively Coupled Plasma Spectroscopy (ICP, SPECTRO, Kleve, Germany) are presented in Supplementary data (Table S1), as well as full XPS analysis (Table S2) and Raman spectra (Figure S1). The distribution of the mean lateral size and specific surface area of the samples are presented in Figures S2 and S3, respectively.

### 3.2. Toxicity Assessment

A table presenting the whole results of the toxicity assessment can be found in Supplementary data (Table S3).

#### 3.2.1. Cytotoxicity (LDH Release Assay)

**Figure 1:**
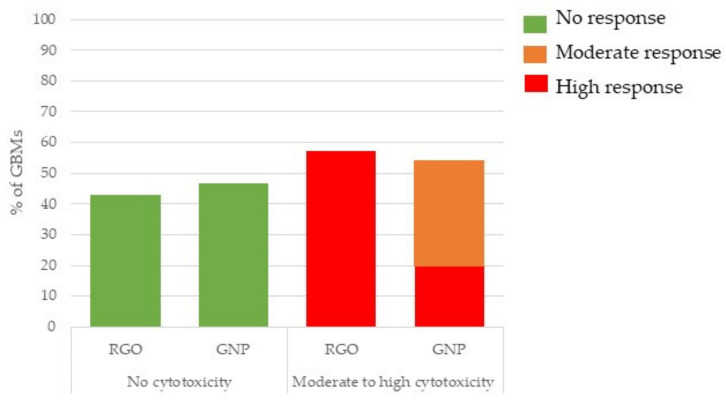
Cytotoxicity classification depending on the GBM type. Three independent experiments were performed, each in quadruplicate and the activity of the released LDH was reported to that of negative control cells (incubated without nanoparticles), then we considered the distribution of GNP and RGO in the groups showing no, moderate or high cytotoxicity.

The cytotoxicity classification depending on GBM type is presented in Figure 1. We separated GNPs and RGOs and studied their toxicity classification, as presented in Section 2.2.7. It appeared that cytotoxicity distribution was not obviously impacted by the GBM type. Also, 43% and 47% of RGOs and GNPs, respectively, did not show cytotoxicity. However, GNPs exhibited mostly moderate cytotoxicity whereas RGOs were mostly highly cytotoxic.

**Figure 2:**
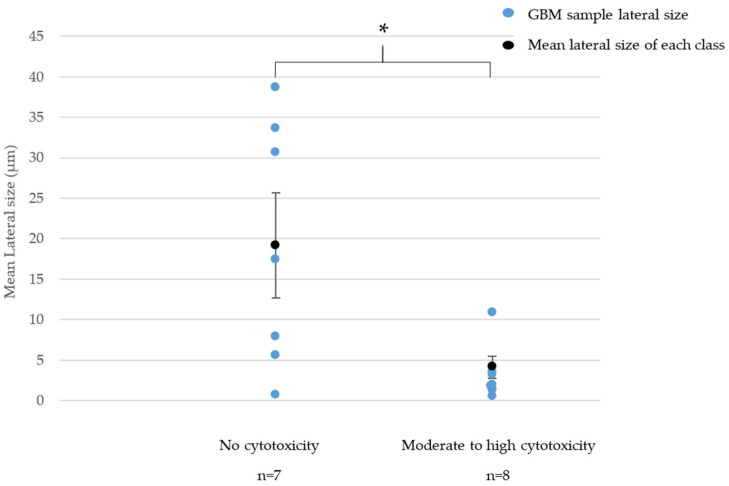
Relationship between cytotoxicity and lateral size for GNPs. * = *p* < 0.05 (Student test).

We then investigated potential correlations between the physicochemical features of the GBMs and their cytotoxicity. A SAR was observed between cytotoxicity and mean lateral size for GNPs, as shown in Figure 2. We did not observe such correlation for RGOs.

In Figure 2, we highlighted a significant difference between the mean lateral size of GNPs classified as non-cytotoxic and the mean lateral size of GNPs classified as moderately or highly cytotoxic. Indeed, samples that were classified as non-cytotoxic had a mean lateral size of 19.2 µm whereas moderately and highly cytotoxic GNPs, respectively, showed a mean lateral size of 4.1 µm and 1.4 µm. Note that non-cytotoxic GNPs had various lateral sizes between 0.7 µm and 38.6 µm. However, the four samples showing a lateral size above 15 µm were all classified as non-cytotoxic.

In summary, RGOs are most likely to be classified as highly cytotoxic whereas GNPs are mostly classified as moderately cytotoxic. For GNPs, the largest (between 15 µm and 40 µm) samples are more likely to be classified as non-cytotoxic whereas the smallest ones (less than 5 µm) are mostly classified as moderately or highly cytotoxic.

#### 3.2.2. Pro-Inflammatory Response (TNF-α ELISA Assay)

**Figure 3:**
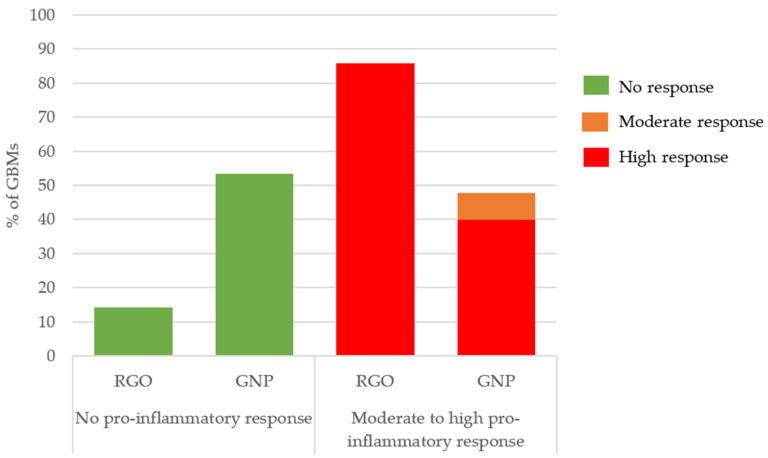
Pro-inflammatory response classification depending on the GBM type. Three independent experiments were performed, each in quadruplicate, and the production of TNF-α was reported to that of control cells (incubated without nanoparticles), then we considered the distribution of GNP and RGO in the groups showing no, moderate or high pro-inflammatory response.

In Figure 3, we observed a different pro-inflammatory response depending on the GBM type. 53% of GNPs and only 14% of the RGOs samples did not induce a pro-inflammatory response whereas 40% of GNPs and 86% of RGOs highly triggered a pro-inflammatory response.

In summary, a vast majority of RGOs caused a high pro-inflammatory response whereas most GNPs caused no pro-inflammatory response. Apart from the impact of the surface chemistry, we did not observe other structure–activity relationships involving the pro-inflammatory response endpoint.

#### 3.2.3. Oxidative Stress

##### ROS Production (DCFH-DA Assay)

**Figure 4:**
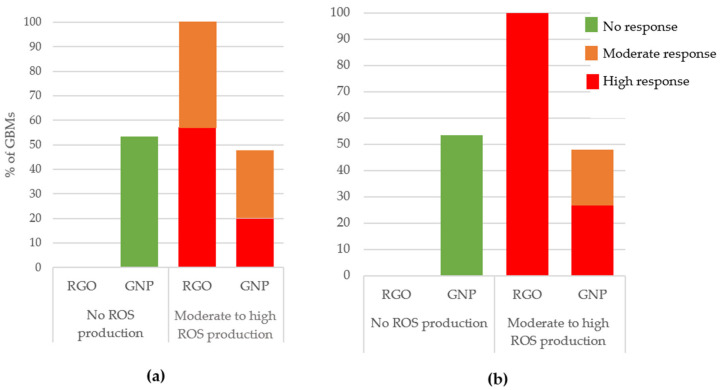
ROS production classification after 90 min (**a**) or 24 h (**b**) of exposure depending on GBM type. Three independent experiments were performed, each in duplicate, and the production of ROS was reported to that of control cells (incubated without nanoparticles), then we considered the distribution of GNP and RGO in the groups showing no, moderate, or high ROS production.

In Figure 4a,b we present the ROS production (90 min and 24 h of exposure) depending on the GBM family. Only GNPs (53% of them for both exposure times) did not induce ROS production. In contrast, most RGOs (57% of them for 90 min exposure time and 100% of them for 24 h exposure time) were classified as causing a high ROS production. These findings highlight a major difference between GNPs and RGOs when it comes to ROS production, demonstrating that, as for cytotoxicity, oxidative stress depends on the chemical nature of the GBMs.

**Figure 5:**
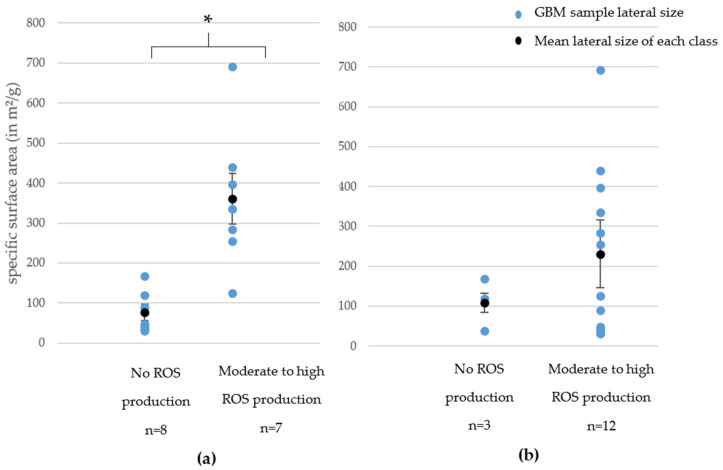
Structure–activity relationship between ROS production after 90 min (**a**) or 24 h (**b**) of exposure and specific surface area. * = *p* < 0.05 (Student test).

We also highlighted an SAR between ROS production at both exposure times and specific surface area for GNPs. This SAR is presented in Figure 5a,b. It appeared that when the SSA increased, the ROS production increased. This trend is particularly clear and statistically significant after a 90 min exposure whereas it appears a little blurred for a 24 h exposure. However, for both exposure times, the samples that were classified as causing high ROS production had higher specific surface areas than samples that caused no ROS production. For RGOs, we did not highlight such correlations.

**Figure 6:**
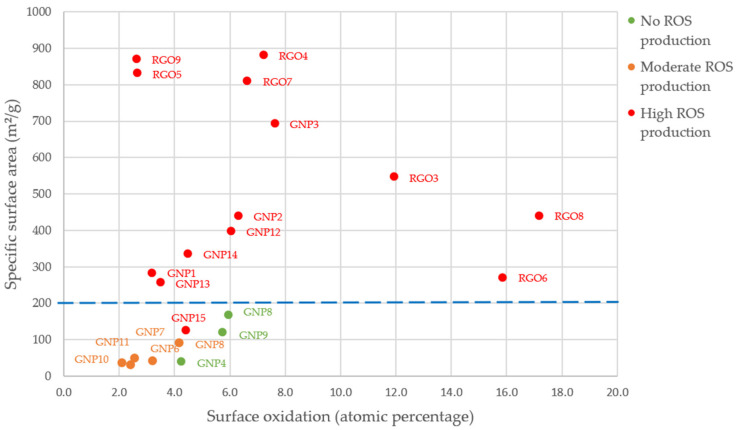
Impact of surface oxidation and specific surface area on ROS production (24-h post-exposure).

In Figure 6, we observed the impact of specific surface area and surface oxidation on ROS production after 24 h of exposure for all GBMs (RGOs and GNPs). We can observe that the three samples showing no impact on ROS production, as well as the five samples that only showed a moderate ROS production after 24 h of exposure, had a specific surface area below 200 m²/g. Among the 14 samples that induced a high ROS production, 13 of them had a specific surface area above 200 m²/g.

For surface oxidation, only three samples showed a surface oxidation of more than 10%. These three samples were also classified as inducing high ROS production. However, we cannot conclude on structure–relationship activity between ROS production and surface oxidation, since most of our samples showed a surface oxidation of less than 8% and variable ROS production.

In summary, a vast majority of RGOs caused a high ROS production whereas most GNPs caused no ROS production. For GNPs, we highlighted SAR between specific surface area and ROS production.

##### Acellular Biological Oxidative Damage (FRAS Assay)

**Figure 7:**
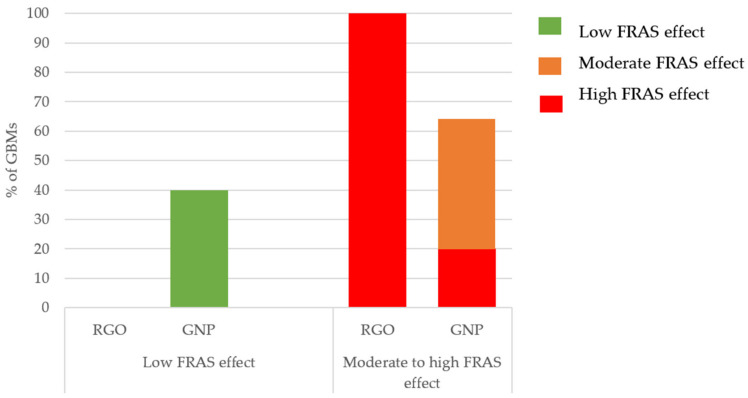
FRAS classification depending on the GBM type. Two independent experiments were performed, each in triplicate and the observed FRAS effect was reported to that of the negative control (serum incubated without nanoparticles), then we considered the distribution of GNP and RGO in the groups showing low, moderate or a high FRAS effect.

For FRAS assay, only GNPs (40% of them for both exposure times) led to a low FRAS effect whereas all RGOs caused a high FRAS effect (Figure 7).

**Figure 8:**
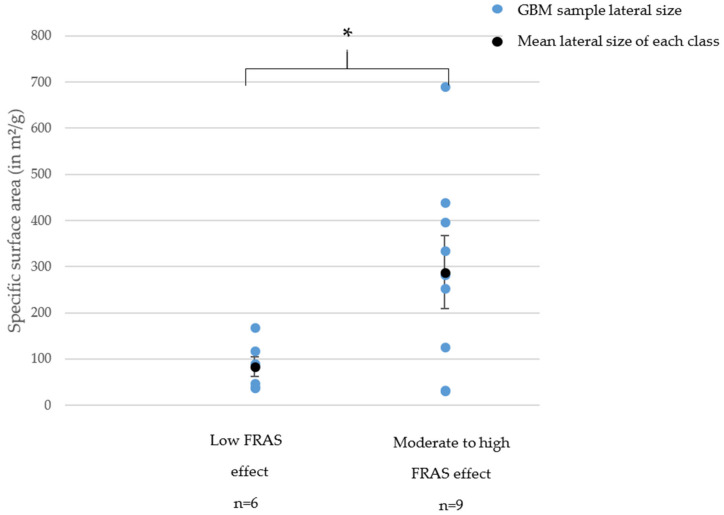
Structure–activity relationship between FRAS effect and specific surface area. * = *p* < 0.05 (Student test).

For this specific endpoint, we observed a structure–activity relationship between SSA and FRAS assay for GNPs (Figure 8).

In summary, all RGOs caused a high FRAS effect whereas GNPs mostly caused a low to moderate FRAS effect. For GNPs, we highlighted a SAR between specific surface area and FRAS effect.

## 4. Discussion

When investigating structure–activity relationships for GBMs, we made the following main findings:

- RGOs and GNPs did not show the same toxicity: RGOs generally appeared to have higher toxicity impacts.
- For GNPs, the cytotoxicity significantly increased when the lateral size decreased.
- For GNPs, the oxidative stress (cellular or acellular) significantly increased when the specific surface area increased, we could note a threshold of 200 m²/g. Below this limit, the samples mostly showed no ROS production and only a low FRAS effect.

We thus found that RGOs tended to show higher toxicity signals, especially for pro-inflammatory response and oxidative stress (ROS production and FRAS assay). These observations are in agreement with several published results. In a study presenting toxicological results on SkinEthic™ (a reconstructed human epidermis) [35], different GBMs including GO (surface oxidation <36.6%, lateral dimension 15,100 nm), RGO (surface oxidation <17.7%, lateral dimension 5500 nm), and FLG, few-layers graphene (surface oxidation <3.7%, lateral dimension of 171 nm) were tested. While FLG and GO did not cause a viability decrease, RGO showed a significant effect. Furthermore, a review [36] classified the toxicity of different GBMs based on a global analysis of in vivo results and highlighted that after inhalation exposure, RGOs caused more damage than GNPs.

These findings can be explained by several parameters. GNPs are composed of at least 10 layers and are not oxidized whereas RGOs are most likely thinner and can sometimes be oxidized. Moreover, their manufacturing process can vary. RGOs are reduced graphene oxide and can be manufactured through various processes. The production involves oxidizing graphite, which will then be sonicated to become graphene oxide. This product will be reduced through various processes that can impact the final product’s toxicity. A study [37] described the toxicological impact of GBMs, including thermally reduced graphene oxide (TRGO) and chemically reduced graphene oxide (CRGO). Their findings showed that TRGO showed a higher toxicity impact on both BEAS-2B and A459 cells for viability, ROS production, genotoxicity, and mitochondria disorder. TRGO was more internalized (through phagocytosis and/or endocytosis) and toxic due to its small lateral dimension (approximately 150 nm vs. 250 nm for CRGO) and its sharp edges. Hence, the production method can have a great impact on GBM’s toxicity: RGO’s manufacturing often involves chemical oxidation followed by a reduction, which can explain the presence of reactive groups on its surface and potentially the generation of carbon radicals, often leading to toxic biological interactions [38]. Moreover, their strong sonication and reduction steps can cause the presence of sharp edges which are well-known to induce higher toxicity for GBMs. This is the conclusion of a study conducted by Akhavan et al. [39] where reduced graphene oxide nanowalls showed higher antibacterial effects than non-reduced graphene oxide nanowalls, due to the sharpness of their edges and their charge transfer, causing damages to bacterial membranes. Our GNPs had ID/IG ratios ranging from 0.06 to 0.72 whereas our RGOs had ID/IG ranging from 0.82 to 1.06. ID/IG ratio is a measurement of surface defects density (the surface defects density increasing with the ID/IG ratio) [40]. We can then conclude that RGOs showed more surface defects than GNPs. This can be due to their manufacturing process which, as described above, is more likely to cause sharp edges and surface defects due to sonication and reduction steps.

The relationship we evidenced between the lateral size and toxicity of GBMs is also in agreement with studies reported in the literature. For instance, it was reported [41] that the smallest graphene (29 nm) had a higher impact than a medium graphene (307 nm) on cytotoxicity (CCK-8). However, the largest graphene (l-G), whose size was unfortunately not measured in this paper, showed cytotoxicity that was comparable to that of the smaller one (s-G). In a second study [42], three GOs were sonicated for increasing time, inducing decreasing lateral size: the non-sonicated one had a large size (1320 nm), the GO sonicated for 2 h generated small (270 nm) particles and the GO sonicated for 26 h generated ultra-small (130 nm) GO. One of the main conclusions was that the more the lateral size was reduced, the more they were internalized. Moreover, the authors described a “mask effect” of these GOs that could facilitate their uptake in the cell, this mask effect being easier for smaller flakes. In another study [43], hMSCs cells were exposed for 24 h to RGOs measuring 11, 90, 418, and 3110 nm. The smallest RGOs showed strong signs of toxicity (cytotoxicity, oxidative stress, and genotoxicity) whereas the largest RGOs only showed very moderate signs of toxicity. On the other hand, in another study [44], three GOs with increasing sizes (from 200 nm to 1025 nm) were tested on a cellular model (J774.A1) and on an in vivo model (mice, exposed through intraperitoneal injection). The largest GO had a higher inflammatory impact compared to the smallest ones on J774.A1 cells, as well as on mice (more pro-inflammatory cytokines could be found in broncho-alveolar lavage, blood serum, and peritoneal lavage in which more inflammatory cells could be found). Overall, the main mechanism was the induction of M1 macrophages through TLR interactions; while the smallest GOs were rapidly taken up by the cells, the largest GOs mostly remained on the macrophage surface and associated with its membrane, activating TLR. Another study [45] compared the toxicity of two GOs measuring less than 200 nm and between 5 and 15 µm. Although both samples were internalized in BEAS-2B cells, the largest caused a higher impact especially on oxidative stress, this trend being verified with in vivo assays. However, please note that in this last study, both samples induced a major cytotoxicity response (measured with LDH assay) from 10 µg/mL. If we transpose these results to ours, both materials would have been classified as highly cytotoxic. In conclusion, the size of the samples appears to be linked to their internalization [46]. GBMs, when they are smaller, seem to be easily internalized, which does not make them systematically more toxic as several publications cited above reported. Indeed, internalization and toxicity impact need to be considered individually even if both will strongly impact GBMs’ biological outcomes. Lastly, the choice of the cell model and even of the model type (in vitro or in vivo) will allow observing various biological impacts that are often due to a combination of internalization and toxicity mode of action.

Smaller GNPs show more sharp edges that could potentially puncture the cells. It has been found that sharp edges of some GBMs cause physical damages to the cell membranes, often leading to cytotoxicity [47].

One may note that the size of the GBMs tested in the publications cited above are not always in the same ranges as the ones tested in our study. The mean lateral size of the samples classified as highly cytotoxic is 1.3 µm, the mean lateral size of the samples classified as moderately cytotoxic is approximately 4 µm and the mean lateral size of the samples classified as non-cytotoxic is around 19 µm whereas, in the literature data, GBMs are mostly measuring 50 to 1000 nm. This is explained by the fact that we focused on commercial GBMs, manufactured for industrial purposes: these samples are often produced at a very high scale, and producing very small GBMs is often an expensive process. Hence, comparing our results with the available literature can be challenging.

Our conclusion stating that lateral size impacts cytotoxicity is in agreement with a machine learning work [48] where SARs were investigated for GBMs. Lateral size was also found to have a major impact on cytotoxicity.

We also found that samples showing the highest specific surface area were mostly classified as highly reactive for oxidative stress (ROS production and FRAS effect). These results are in agreement with a few publications. Unfortunately, the specific surface area is rarely explored for GBMs being tested for their toxicity and we had only a few of them studying GBMs with measured SSA for comparable toxicity endpoints. We considered two different papers [49,50] presenting the results of MTT assessment for human respiratory cells (BEAS-2B and A549). The samples tested in each study were both GNPs, mostly differing by their specific size area (196 m²/g vs. 735 m²/g). It appeared that the GNP with a SSA of 735 m²/g showed much higher cytotoxicity than the sample with a SSA of 196 m²/g (after 24 h of exposure). Unfortunately, the sample with a SSA of 196 m²/g was not tested for oxidative stress. However, the sample with a SSA of 735 m²/g was tested with DCFH-DA assay, and its ROS production was high: after 24 h of exposure, a dose of 5 µg/mL was enough to significantly induce ROS production.

However, a point concerning the pore of the samples needs to be underlined: in this study, we did not assess the pore size of our samples. This information can yet impact GBMs’ bioavailability and toxicity [51].

For in vitro testing, it appeared that samples showing the highest specific surface area had the highest toxicity impacts. This can easily be explained by the highest available surface for biological interaction, often leading to more toxicity [52]. In vivo inhalation studies [53,54] were also performed on rats using various samples with increasing SSA from 8 to 131 m²/g. It appeared that the GBMs having the smallest SSA showed a lower pulmonary impact (proteins and cells in broncho-alveolar lavage). On the contrary, another in vivo study [55] used GNPs Gr20, Gr5, and Gr1 which had increasing sizes from 20 µm to less than 2 µm and are still respirable for humans. The Gr1 had the most reactive surface (DTT) due to its high SSA (735 m²/g vs approximately 100 m²/g for Gr5 and Gr20), but Gr20 appeared to cause a higher lung inflammation after a pharyngeal aspiration on mice. Moreover, the BAL was collected and a LDH quantification was made and the Gr20 caused a higher LDH release than the Gr1. Gr20 and Gr5 had high aspect ratios, possibly involving frustrated phagocytosis (even if no obvious mark of this mechanism was observed), which might have caused a higher effect than the increasing specific surface area.

Oxidative stress is a major mode of action for GBMs [56] and often impacts many other mechanisms such as mitochondria activity [57] that can eventually lead to apoptosis [58]. The complexity of oxidative stress and its implication in cell physiology can make the study of potential SARs even more challenging. Hence, studying SARs involving oxidative stress for GBMs, must be done carefully while considering the whole organism.

To conclude, our findings should be considered not only at a scale of one cell but also of a whole organism. When samples are tested on in vivo models, the ones showing high SSA are still more likely to have the highest biological interactions, possibly leading to more toxicity impacts. Nevertheless, this fact has yet to be put into perspective with the fact that other characteristics might be involved in in vivo toxicity. For inhalation exposure, pulmonary clearance is an essential parameter and needs to be considered for an organism-scale impact. Indeed, it can be modulated by several parameters such as surface chemistry or size which will determine if GBMs will be easily cleared from the lungs. This parameter is critical and will strongly affect their global toxicity but does not involve the same mechanisms as in vitro toxicity.

In our study, we mostly spotted SARs for GNPs. This can be explained by the fact that RGOs, being more reactive, are more likely to be mostly classified in the highest toxicity categories. Hence, we recommend using the lowest exposure doses for reactive samples such as RGOs (our lowest dose was 15 µg/mL and many RGOs showed a significant impact from this dose) to be able to classify them in various toxicity classes. Moreover, we only tested seven RGO samples, which appears to be a little bit low to conclude on significant correlations. We suggest studying at least 10 samples of each GBMs family to obtain relevant conclusions.

We observed that lateral size was correlated to cytotoxicity: the smallest GBMs caused significantly higher cytotoxicity. This might be linked to their internalization, as previously discussed. However, we also know that an easier internalization does not systematically lead to higher cytotoxicity. A study of the internalization might allow having clearer insight into the GBMs’ mode of action and how a smaller lateral size could lead to higher cytotoxicity.

We also have to keep in mind that physicochemical parameters can sometimes be correlated with each other: this is particularly the case for specific surface area and lateral size of GBMs. In Figure S4 (Supplementary Materials), we propose an analysis of the correlation between specific surface area and lateral size. We concluded that for GBMs smaller than 5 µm, it appeared to be a correlation between specific surface area and lateral size: when lateral size increased, the specific surface area decreased, which was expected. However, GBMs higher than 5 µm showed various specific surface areas that did not appear to follow the same trend. We can conclude that even if the intercorrelation between physicochemical parameters is an important fact that must be taken into account especially for SARs study, the nature and stability of their correlation is sometimes unexpected. Assessing the parameters remains the safest way to establish a relevant physicochemical profile of each sample.

The tested samples were various GBMs but none of them was functionalized. Surface chemistry and functionalization are yet well-known to strongly impact toxicity [59]. This aspect should be explored in prospective studies to integrate the impact of functionalization in GBMs’ SARs.

For our SARs, we highlighted that our results are in agreement with several studies and can be mechanistically explained. Nevertheless, other studies (especially in vivo studies) sometimes found opposite SARs. In toxicology, the choice of the model is critical and can strongly influence the outcome: with in vitro testing, results can sometimes vary depending on the cell type or the assay [51]. When we compare in vivo and in vitro testing, the difference is even more obvious [52].

For risk assessment and study of SARs, both models present their qualities: in vitro studies allow to test a lot of samples quicker, with minimum bias, and allow precise observations at a cell scale. In vivo models allow observations with an exposure that can be more easily transposed to humans, and a global insight of the toxic effects at an organism scale. However, SARs studies require testing a great number of samples to highlight significant correlations that could be the basis for the construction of safe-by-design GBMs. For that reason, we recommend using in vitro models for SARs studies, considering that the same cell model should be used for the whole testing to avoid bias. In vivo studies are extremely relevant but, since they should be used as rarely as possible, they should only be used to confirm the conclusions of the SARs studies.

With our study, we propose the first examination of potential SARs. Firstly, RGOs appear more toxic than GNPs. That can be explained by their production process that can sometimes lead them to have the sharpest edges, more surface defects, and more chemically reactive surfaces. For GNPs, we concluded that the cytotoxicity was linked to lateral size: the smaller (around 1 µm) GNPs were, the more cytotoxic they were whereas the largest GNPs, around 4 µm and 19 µm, respectively, showed moderate cytotoxicity or none. Even though additional studies are necessary to confirm this hypothesis, this might be explained by the internalization process that appears to be more effective for smaller GNPs. Lastly, we highlighted that for GNPs, the highest specific surface area often led to the highest oxidative stress impact. This is probably due to the fact that the highest available surface often allows more biological interactions.

## 5. Conclusions

To our knowledge, this study is the first one proposing a complete and systematic physicochemical characterization and in vitro toxicity assessment of a large pool of different GBMs. This methodology enabled us to compare the results with no risk of bias due to the cell model or assay variation. By exploring a large set of physicochemical characteristics of various GBMs, we highlighted quite clear structure–activity relationships. We concluded that GNPs with the smallest lateral size (approximately 1 µm) were the more cytotoxic whereas largest GNPs (more than 10 µm) were more likely to be non-cytotoxic. We also spotted a clear relationship between specific surface area and oxidative stress (ROS production and FRAS effect). Lastly, we observed that RGOs were most likely to cause a high pro-inflammatory response and ROS production when compared to GNPs. These different biological impacts between RGOs and GNPs stress the importance of considering each family of GBMs individually when studying their toxicity. The evidenced structure–activity relationships could serve as a basis for a safe-by-design approach, allowing to lower the toxicity of GBMs of great interest for several industrial fields.

## Figures and Tables

**Table 1:** Physicochemical features of the 22 GBMs, 2 carbon black and 1 amorphous silica.

	Surface Oxidation (% O)	Mean Lateral Size (µm)	Surface Defects (ID/IG)	Specific Surface Area (m²/g)
GNP1	3.2	1.25	0.369	283
GNP2	6.3	0.66	0.470	439
GNP3	7.6	0.53	0.724	692
GNP4	4.3	3.56	0.340	38
GNP5	3.3	5.58	0.062	41
GNP6	2.6	7.91	0.146	48
GNP7	4.2	10.86	0.101	89
GNP8	5.9	17.34	0.634	168
GNP9	5.7	38.57	0.068	119
GNP10	2.1	33.54	0.132	34
GNP11	2.5	30.70	0.225	31
GNP12	6.1	1.63	0.348	396
GNP13	4.4	3.16	0.645	125
GNP14	4.5	1.51	0.346	335
GNP15	3.5	2.02	0.321	255
rGO3	11.9	8.26	1.038	545
rGO4	7.2	31.56	0.937	880
rGO5	2.7	6.99	1.066	830
rGO6	15.9	32.01	0.905	270
rGO7	6.7	15.1	0.957	810
rGO8	17.2	1.04	0.908	440
rGO9	2.6	1.11	1.066	870
CB1	2.6	0.36	NA	112
CB2	2.3	0.9	NA	85
Amorphous Silica	70.0	0.09	NA	160

## Data Availability

The data is available on reasonable request from the corresponding author.

## Supplementary Materials

The following are available online at https://www.mdpi.com/article/10.3390/nano11112963/s1, Table S1: ICP analysis, Table S2: full XPS analysis, Figure S1: Raman spectra (a) RGOs and (b) GNPs, Table S3: Toxicological studies results. Figure S2: Mean lateral size distribution. Figure S3: Specific surface area distribution. Figure S4: Study of intercorrelation of physicochemical characteristics (specific surface area and lateral size).

## Abbreviations

µm: micrometer. BET: Brunauer, Emmett, and Teller. CCK-8: Cell Counting Kit-8. CRGO: chemically reduced graphene oxide. DCFH-DA: 2′,7′–dichlorofluorescin diacetate. DMEM: Dulbecco’s Modified Eagle Medium. FRAS: Ferric reducing ability of the serum. GBM: graphene-based material. GNP: graphene nanoplate. GO: graphene oxide. GQD: graphene quantum dots. hMSCs: Human Mesenchymal Stem Cells. ICP: Inductively Coupled Plasma. LDH: lactate deshydrogenase. LOAEL: Lowest Observed Adverse Effect Level. MTT: tetrazolium salt (3-(4,5-dimethylthiazol-2-yl)-2,5-diphenyltetrazolium bromide. nm: nanometer. RGO: reduced graphene oxide. ROS: reactive oxygen species. SAR: structure–activity relationship. SSA: specific surface area. TLR: toll-like receptor. TNF-α: tumor necrosis factorα. TRGO: thermally reduced graphene oxide. XPS: X-ray spectroscopy.
