# Supplementary material for: Structure–Activity Relationship of Graphene-Based Materials: Impact of the Surface Chemistry, Surface Specific Area and Lateral Size on Their In Vitro Toxicity
Source: Nanomaterials (Basel). 2021 Nov 4;11(11):2963. doi: 10.3390/nano11112963 (PMC8619174; doi:10.3390/nano11112963)

Supplementary data

Table S1: ICP analysis

	ICP results (ppm)																					
	Al	B	Ba	Ca	Co	Cr	Cu	Fe	K	Li	Mg	Mn	Mo	Na	Ni	S	Si	Sn	Sr	Ti	V	Zn	Zr
GNP1	54	16	1	90	0	2	2	21	47	0	45	0	7	40	0	44	0	4	0	20	4	32	13
GNP2	430	92	3	538	0	1	5	1008	201	0	304	13	2	162	5	136	0	368	1	34	8	12	3
GNP3	434	97	5	147	0	26	21	589	708	0	182	6	19	420	22	421	0	32	2	108	33	8	13
GNP4	169	76	2	78	0	300	17	1015	403	0	29	32	15	162	81	2346	0	13	1	293	33	33	8
GNP5	113	58	1	40	0	275	7	1279	233	0	37	32	9	168	136	1472	0	14	0	5	9	23	5
GNP6	108	115	1	33	0	350	9	1594	196	0	37	45	9	188	175	1910	0	9	0	33	9	12	4
GNP7	87	45	1	20	0	222	5	1044	265	0	38	42	5	197	91	6464	0	9	0	138	12	13	2
GNP8	118	21	1	42	0	201	9	1068	223	0	80	29	3	135	90	12114	0	10	0	70	6	15	4
GNP9	117	61	1	122	0	231	5	1224	299	0	61	34	4	145	100	9431	0	13	0	34	7	23	3
GNP10	86	5	1	63	0	214	4	1270	173	0	36	42	4	319	100	1420	0	17	0	8	7	13	2
GNP11	184	16	1	83	0	267	8	1149	134	0	97	52	4	173	101	2085	0	6	0	5	4	22	1
GNP12	197	42	3	784	0	3	154	5980	189	0	207	29	4	73	32	3727	0	9	2	9	6	17	2
GNP13	119	72	1	65	0	0	0	524	137	28	4	5	0	76	8	20	0	7	1	16	127	22	2
GNP14	85	41	4	235	0	7	8	2373	114	0	28	19	4	47	6	28	0	9	2	23	11	30	1
GNP15	87	54	12	203	0	0	0	1173	121	0	24	6	3	46	2	44	0	9	5	35	7	16	3
rGO3	236	76	1	135	0	54	3	252	393	0	30	2058	3	98	24	156	0	307	1	3	5	221	2
rGO4	279	155	4	1315	0	7	0	151	1907	0	1257	299	66	4518	77	14679	0	14	14	3	11	599	7
rGO5	269	230	2	1661	0	64	0	215	1035	0	1369	431	15	1618	34	2819	0	196	15	68	12	55	56
rGO6	372	212	10	668	0	13	0	557	2403	0	585	1479	169	3089	658	4440	0	17	7	6	11	146	3
rGO7	235	239	3	1416	0	0	0	95	1866	0	1360	457	111	5893	53	9714	0	15	14	17	7	35	3
rGO8	463	426	3	338	0	34	9	224	1512	0	209	1907	27	862	36	5141	0	36	3	567	52	34	12
rGO9	402	165	2	256	0	23	0	215	1505	0	235	1847	23	744	38	761	0	73	2	676	51	66	7
Amorphous Silica	3223	43	19	388	0	0	0	62	90	0	49	1	2	7669	0	3356	0	5	1	24	2	7	6
CB1	136	15	1	411	0	2	0	66	221	0	152	1	0	667	1	10799	0	5	5	2	2	29	0
CB2	92	11	1	286	0	1	0	46	216	0	97	1	0	101	1	9875	0	7	1	3	0	20	0

Table S2: XPS analysis

	XPS (atomic %)				
	C	O	N	S	Si
GNP1	96,8	3,2	0,0	0,0	0,0
GNP2	93,7	6,3	0,0	0,0	0,0
GNP3	92,4	7,6	0,0	0,0	0,0
GNP4	95,7	4,3	0,0	0,0	0,0
GNP5	96,7	3,3	0,0	0,0	0,0
GNP6	97,3	2,6	0,0	0,1	0,0
GNP7	95,4	4,2	0,0	0,4	0,0
GNP8	93,2	5,9	0,5	0,4	0,0
GNP9	93,2	5,7	0,7	0,3	0,0
GNP10	97,9	2,1	0,0	0,0	0,0
GNP11	97,5	2,5	0,0	0,0	0,0
GNP12	93,2	6,1	0,8	0,0	0,0
GNP13	95,6	4,4	0,0	0,0	0,0
GNP14	94,9	4,5	0,6	0,0	0,0
GNP15	96,0	3,5	0,5	0,0	0,0
rGO3	88,1	11,9	0,0	0,0	0,0
rGO4	91,7	7,2	0,0	1,0	0,0
rGO5	96,6	2,7	0,6	0,2	0,0
rGO6	84,1	15,9	0,0	0,0	0,0
rGO7	92,6	6,7	0,0	0,7	0,0
rGO8	82,8	17,2	0,0	0,0	0,0
rGO9	95,2	2,6	2,2	0,0	0,0
Amorphous Silica	96,9	2,6	0,0	0,5	0,0
CB1	97,2	2,3	0,0	0,5	0,0
CB2	3,6	70,0	0,0	0,0	26,4

Figure S1: Raman spectra for RGOs (a) and for GNPs (b)

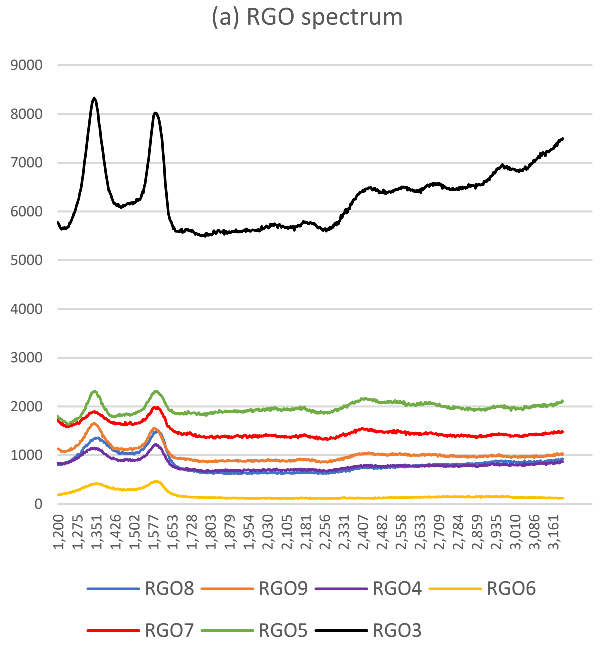

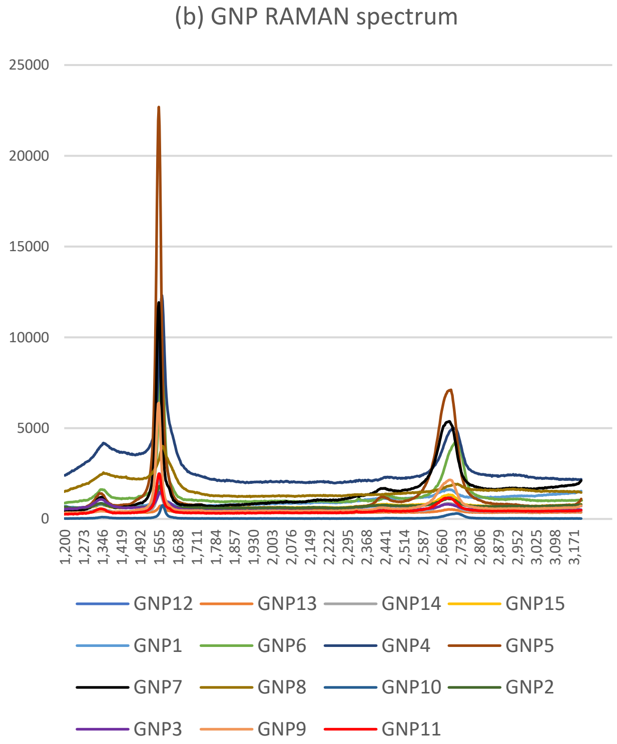

Figure S2: Mean lateral size distribution

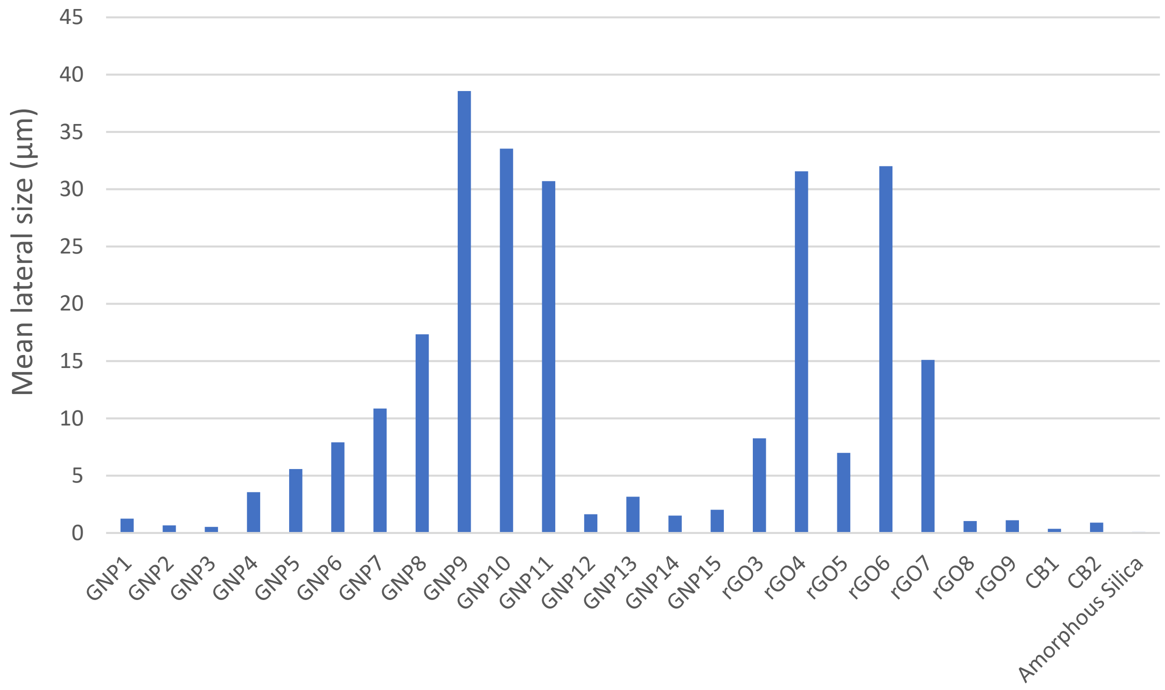

Figure S3: Specific surface area distribution

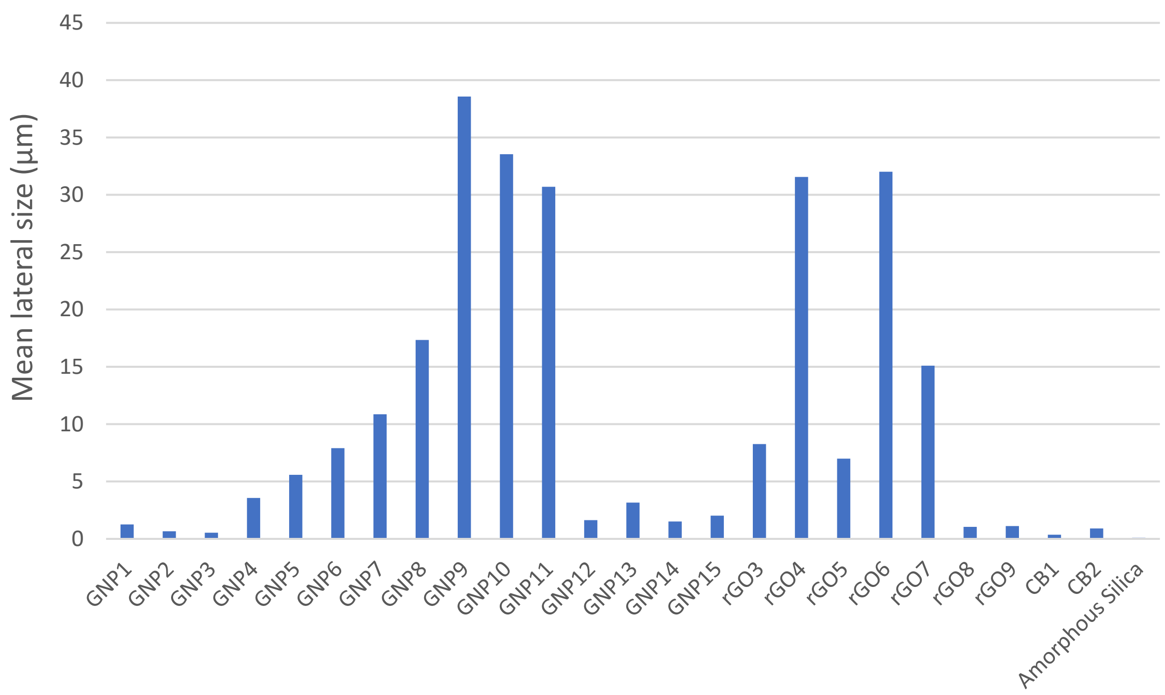

Table S3: Toxicology results

Please note that the number indicated in each cells are a fold compared to negative control. 1 means the difference was not significant. For the FRAS assay, the results are expressed in BOD units (biological oxidative damage), with a TEU (trolox equivalent unit) calibration, in mMTEU. For the statistical analysis, we used a Dunett's multiple comparison test performed on Graphpad Prism software.

	Cytotoxicity (24h)				Inflammation (24h)				Oxidative stress (90min)				Oxidative stress (24h)				FRAS (3h)		
	15 µg/mL	30 µg/mL	60 µg/mL	120 µg/mL	15 µg/mL	30 µg/mL	60 µg/mL	120 µg/ml	15 µg/mL	30 µg/mL	60 µg/mL	120 µg/mL	15 µg/mL	30 µg/mL	60 µg/mL	120 µg/ml	5g/L	10g/L	20g/L
**GNP1**	1	1	1	3.5	1	1	2.5	5.3	1	1	3.8	4.9	8.6	11.8	18.5	30.2	76	92	100
**GNP2**	1	1	1	1	1	1	2.6	5.6	1	1	5.7	8.7	15.5	20	24.5	32.7	77	98	102
**GNP3**	2.61	1	3.3	4.7	1	1	1.8	4	2.6	3.8	5.5	9.7	13.5	19.4	20.9	22.8	90	101	109
**GNP4**	1	1	1	2.8	2.6	4.8	7.8	9.9	1	1	1	1	1	1	1	1	9	30	46
**GNP5**	1	1	1	1	2.7	2.7	3	3.7	1	1	1	1	1	1	1	10.8	26	40	71
**GNP6**	1	1	1	1	2.3	3.4	5.1	7	1	1	1	1	1	1	1	21.9	21	38	68
**GNP7**	1	1	1	3	2.4	3	3.2	3.5	1	1	1	1	1	1	1	11.5	16	40	65
**GNP8**	1	1	1	1	1	1	2.8	3	1	1	1	1	1	1	1	1	4	30	45
**GNP9**	1	1	1	1	1	1	2.4	2.8	1	1	1	1	1	1	1	1	17	35	56
**GNP10**	1	1	1	1	1	1	1	1	1	1	1	1	1	1	1	15.9	46	75	91
**GNP11**	1	1	1	1	1	1	1	1	1	1	1	1	1	1	1	8.8	42	74	92
**GNP12**	1	1	2.4	3.5	1	2.7	4.9	6.4	1	2.8	4.5	8.1	9.8	12.9	22.6	26.3	54	81	95
**GNP13**	1	2.6	3.3	4.6	2.5	4.2	6.3	7.1	1	1	3.3	6.5	1	12.8	22.8	35.1	43	68	89
**GNP14**	1	2.6	2.7	4.6	2.3	4	6	6.5	1	2.7	4.6	7.2	1	13.6	22.5	30.6	55	85	97
**GNP15**	1	1	1	3.6	1	1	4.1	5.9	1	1	1	3.7	1	10	18.3	24.4	36	60	72
**RGO3**	1	1	1	1	1	1	3.2	6.4	1	1	2.6	3.8	1	10.3	15.5	21.5	87	98	101
**RGO4**	1	2.4	2.4	2.5	7.1	9.1	10.1	10	2.5	3.4	3.6	4.3	16.9	17.6	18.7	17.1	65	80	**NA**
**RGO5**	1	1	1	1	5.4	6.8	9	9.2	1	1	2.7	2.9	23.3	20.9	25.9	21.5	59	100	**NA**
**RGO6**	1	1	1	1	2.9	3.8	4.7	5	2.5	3.1	4.7	6.5	22.5	24.1	33.9	42.7	72	96	**NA**
**RGO7**	2.8	3.2	3.3	3.3	10.8	10.7	11	10.8	2.7	3.4	4.3	4	20.9	18.3	21.2	14.1	75	92	**NA**
**RGO8**	4.2	4.9	4.7	5	5.9	6.2	6	5.9	1	1	2.6	3.7	9.8	13.5	19.4	28.3	80	97	**NA**
**RGO9**	4.6	5.4	5.4	6	6.2	6.1	6.3	6.2	9.1	10.7	15.6	8.7	31.1	23.6	24	15.3	93	103	**NA**
**CB1**	1	1	1	2.9	1	2.7	4.7	6.9	1	1	1	2.6	9.5	10.2	17.4	20.1	5	3	22
**CB2**	1	1	1	1	1	1	2.3	4.2	1	2.9	3.5	7	9.4	12.9	19.4	33.2	11	19	29
**Amorphous silica**	1	2.6	3.6	4.6	1	2.2	3.8	5	1	1	1	1	1	1	1	1	3	5	7

Figure S4: Correlation between specific surface area and mean lateral size.

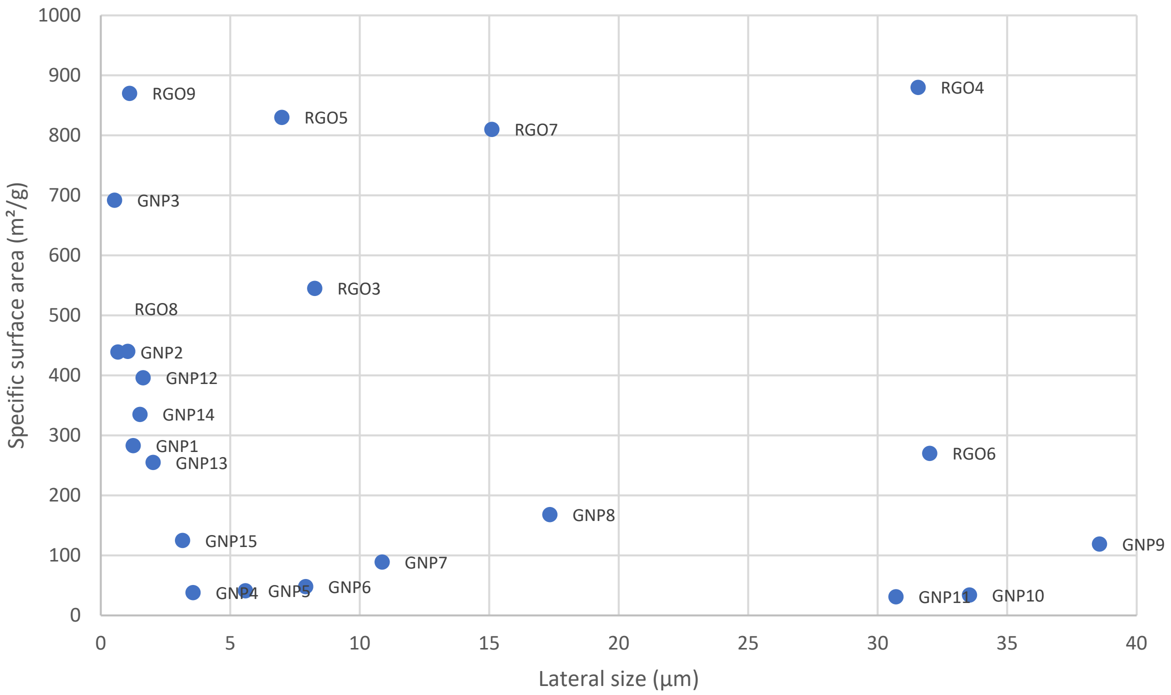

Supplement: Supplementary file 1 [file nanomaterials-11-02963-s001.zip › nanomaterials-1438696-supplementary.pdf]
